# Deep Sequencing Analyses of Low Density Microbial Communities: Working at the Boundary of Accurate Microbiota Detection

**DOI:** 10.1371/journal.pone.0032942

**Published:** 2012-03-06

**Authors:** Giske Biesbroek, Elisabeth A. M. Sanders, Guus Roeselers, Xinhui Wang, Martien P. M. Caspers, Krzysztof Trzciński, Debby Bogaert, Bart J. F. Keijser

**Affiliations:** 1 Department of Pediatric Infectious Diseases and Immunity UMC Utrecht, Utrecht, The Netherlands; 2 Research Group Microbiology and Systems Biology, TNO Earth, Environmental and Life Sciences, Zeist, The Netherlands; Argonne National Laboratory, United States of America

## Abstract

**Introduction:**

Accurate analyses of microbiota composition of low-density communities (10^3^–10^4^ bacteria/sample) can be challenging. Background DNA from chemicals and consumables, extraction biases as well as differences in PCR efficiency can significantly interfere with microbiota assessment. This study was aiming to establish protocols for accurate microbiota analysis at low microbial density.

**Methods:**

To examine possible effects of bacterial density on microbiota analyses we compared microbiota profiles of serial diluted saliva and low (nares, nasopharynx) and high-density (oropharynx) upper airway communities in four healthy individuals. DNA was extracted with four different extraction methods (Epicentre Masterpure, Qiagen DNeasy, Mobio Powersoil and a phenol bead-beating protocol combined with Agowa-Mag-mini). Bacterial DNA recovery was analysed by 16S qPCR and microbiota profiles through GS-FLX-Titanium-Sequencing of 16S rRNA gene amplicons spanning the V5–V7 regions.

**Results:**

Lower template concentrations significantly impacted microbiota profiling results. With higher dilutions, low abundant species were overrepresented. In samples of <10^5^ bacteria per ml, *e.g.* DNA <1 pg/µl, microbiota profiling deviated from the original sample and other dilutions showing a significant increase in the taxa Proteobacteria and decrease in Bacteroidetes. In similar low density samples, DNA extraction method determined if DNA levels were below or above 1 pg/µl and, together with lysis preferences per method, had profound impact on microbiota analyses in both relative abundance as well as representation of species.

**Conclusion:**

This study aimed to interpret microbiota analyses of low-density communities. Bacterial density seemed to interfere with microbiota analyses at < than 10^6^ bacteria per ml or DNA <1 pg/µl. We therefore recommend this threshold for working with low density materials. This study underlines that bias reduction is crucial for adequate profiling of especially low-density bacterial communities.

## Introduction

Deep sequencing techniques allow for detailed analyses of microbial communities that occupy skin and various mucosal sites of the human body and exploration of their potential role in health and disease. Bacterial composition differs greatly between body sites and between individuals, depending on host and environmental parameters such as nutrient availability, humidity, mucosal structure and immune status [1], [2], [3], [4]. Not only microbial composition and dynamics but also community density varies greatly per site, e.g. 10^11^–10^12^ bacteria/g in fecal material [5] to only 10^4^–10^5^ bacteria/cm^2^ in the nasopharyngeal region [6]. Bacterial density is important for quorum sensing and cross talk between bacteria, in which it determines differential gene regulation and subsequent the particular behavior of bacteria. By this cross-communication bacteria can regulate virulence factor production and metabolic demands of the community they live in [7], [8].

The upper airway is the port d'entrée for infections and insight into microbial community structures in these sites could contribute to our understanding of pathogenesis of respiratory infections. Most of these niches, such as the nasopharynx, are colonized at low density. Furthermore, individuals can vary greatly in colonization density of the same niche, possibly reflecting physicochemical differences. For comprehensive and accurate insight in the microbiota of these low-density regions, and inter-individual comparison, understanding the effect of low bacterial 16S gene template concentrations on deep sequencing analyses is relevant, especially since most studies have been focusing on bacterial habitats, where bacterial density, composition and diversity is different from these habitats e.g. gut microbiota [5], [9], [10], [11].

We therefore studied the effect of bacterial density on microbiota analyses by 16S rDNA pyrosequencing of serially diluted saliva. To adjust for possible DNA extraction biases, we extracted DNA by four commonly used DNA extraction methods. To be able to extrapolate the dilution results to the natural situation we compared 16S rDNA gene pyrosequencing-based results for low-density (nares, nasopharynx) and high-density communities (saliva, oropharynx) of the upper respiratory tract of four healthy individuals.

## Results

### Bacterial density in nasopharyngeal samples

During a vaccine intervention trial, nasopharyngeal swabs were collected in 1003 infants during the first 24 months of their life [12]. This sample collection enabled us to gain insight in the dynamics of nasopharyngeal microbiota composition in relation to pneumococcal vaccination and other epidemiological factors. However, to enable analysis of the temporal dynamics of the nasopharyngeal microbiota, unbiased microbiome analysis of the swab collection is essential. In a previous reported study [6] 16SrDNA levels of 154 randomly selected nasopharyngeal swabs of this collection ranged between <0.5 pg/µl to o.12 ng/µl with an average of 7.4 pg/µl (Figure S1). In 45% of samples, DNA levels were between 1 and 10 pg/µl , in 35% less than 1 pg/µl and 19% above 10 pg/µl. Although symptoms of a common cold appeared to be associated with higher DNA levels in the swabs (data not shown), we were not able to identify this or other biological factors attributing to the large variation in DNA content, although differences in sampling efficiency may play a role. These results, however, prompted us to investigate the effects of DNA template concentration on accurate 16 s rDNA microbiota profiling and to establish a protocol to correctly assess these low abundant regions taking into account possible interfering biases due to technical analyses.

### Effect of bacterial density on microbiota composition

To elucidate the effect of bacterial density on the comparability of 454 prosequencing analyses, we designed a titration experiment using saliva from one person with known bacterial cell density of 10^9^ and made a dilution series corresponding to 10^7^ to 10^5^ bacteria per ml (named dilution 1 to 3 respectively, table 1). From each sample, DNA was extracted by four different extraction methods e.g. Epicentre Masterpure DNA purification kit, Qiagen DNeasy Blood & Tissue Kit, MoBio PowerSoil DNA Extraction Kit or a phenol/bead beating method combined with the Agowa Mag mini DNA extraction kit, hereafter referred to as Epicentre, Qiagen, Mobio and Agowa respectively. Using these four extraction methods reduced the potential bias introduced by mechanism of lysis or DNA capturing. The microbiota of the samples was subsequently analysed by 16srDNA pyrosequening.

**Table 1:** Sequence characteristics of undiluted and serially diluted saliva (1 individual), isolated with the Agowa extraction method.

	Undiluted	Dilution 1	Dilution 2	Dilution 3
**quantity by RT-PCR (in pg/µl)**	8,39E+03	6,12E+01	9,75	8.42E-01
**# all sequences**	4328	5719	4744	11871
**# total of sequences after quality (% of total)**	1668 (38)	2306 (40)	2119 (45)	4249(36)
-**# removed sequences < quality 35** * **(%)**	1985 (45.9)	3265 (57.1)	2501 (52.7)	7181 (60.5)
-**# removed chimeras (%)**	252 (5,8)	137 (2,4)	49 (1,0)	300 (2,5)
-**# unique sequences (%)**	366 (22)	512 (22)	474 (22)	1361 (32)
**Normalized to (#sequences)**	1000	1000	1000	1000
**- #unique sequences**	82	67	75	112
**Coverage** §	0,96	0,98	0,97	0,97
**Shannon diversity index**	2,62	2,7	3,07	3,45
**Simpson diversity index**	0,16	0,13	0,08	0,07

A clear increase in unique sequences, unclassified bacteria and Shannon diversity index was seen in dilution 3 (10^5^ bacteria/ml). Undiluted = undiluted saliva, 1 = dilution 1, 10^7^ bacteria/ml, 2 = dilution 2, 10^6^ bacteria/ml and 3 = dilution 3, 10^5^ bacteria/ml.

* Using a moving window 50 bp long to check that the average quality score over the region does not drop below 35 (using the qual file).

§ The **coverage** calculator returns Good's coverage for an OTU definition.

**Figure 1:**
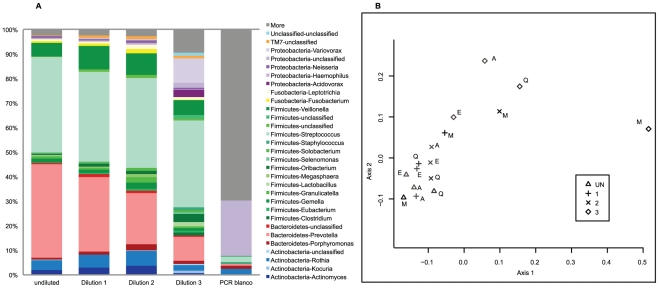
Microbiota composition and Principal Coordinate of Analyses for serially diluted saliva. *a*. Microbiota composition for undiluted and serially diluted saliva of individual 1 isolated with the Agowa method. For undiluted saliva, the dilutions and the PCR blank, relative abundance of the genera expressed in percentages are shown on the y-axes. The legend shows the 30 most abundant taxa and genera found in colors. Microbiota composition starts to deviate from the original sample at dilution 3 (10^5^ bacteria/ml). In dilutions 1 and 2 low abundant genera seemed to increase in abundance, while high abundant genera decrease in abundance. *b*. Unweighted UniFrac Principal Coordinate Analyses plot of undiluted and serially diluted saliva isolated with four DNA extraction methods. Great overlap in sequence representation was seen between undiluted samples and samples diluted up to dilution 2 (10^6^ bacteria per ml) for all DNA extraction methods except for Mobio. Dilution 3 samples (10^5^ bacteria per ml) are deviating from the original sample. DNA extraction methods are depicted in characters (E = Epicentre, M = Mobio, Q = Qiagen and A = Agowa). The dilutions are depicted in symbols as shown in the legend. Undiluted = undiluted saliva, 1 = dilution 1, 10^7^ bacteria/ml, 2 = dilution 2, 10^6^ bacteria/ml and 3 = dilution 3, 10^5^ bacteria/ml.

Unweighted Unifrac analysis of the 454 sequence data revealed great concordance in microbiota composition between undiluted samples and samples diluted up to 10^6^ bacteria per ml for all DNA extraction methods with the exception of Mobio extracted samples (figure 1b and S3). Contrastingly, weighted unifrac analysis, taken into account abundance, revealed that decreasing template levels (increasing dilution) coincided with significant decrease in the levels of dominant Bacteroidetes species, e.g. *Prevotella*, and concomitant increasing abundance of minor Firmicutes genera, e.g. *Veilonella* (Figure 1a and S2).

**Figure 2:**
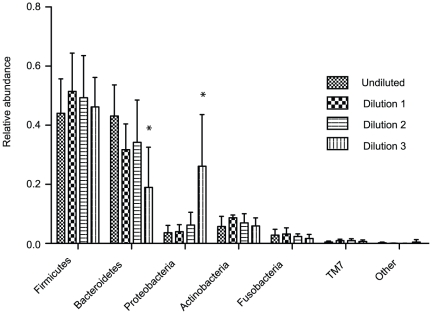
Average relative abundance of the 6 main taxa in undiluted saliva and dilutions 1 to 3 (10^7^ to 10^5^ bacteria per ml, respectively). Shown in error bars is the standard deviation per dilution indicative of the variation between DNA extraction methods. We used ANOVA statistics to test for significant differences. Dilution 3 shows a significant increase in Proteobacteria (p<0.001, mean 26,11% and 3.6%, SD 17.5 and 2.5% respectively) and a significant decrease in Bacteroidetes (p<0.001, mean 18.99% and 43.1% respectively) compared to the undiluted saliva samples. By diluting the sample up to 10^5^ bacteria per ml an increase in Firmicutes, mostly *Veilonella*, was observed, and a decrease in Bacteroidetes, mostly *Prevotella*.

Furthermore, samples diluted below a level of 10^6^ bacteria per ml (dilution 3, overall DNA template below 1 pg/µl) displayed a clear shift in unweighted UniFrac distance, suggesting significant impact on species composition (Amova <0.001, Fig. 1b). Bacterial sample concentration corresponded to 30.97% of the variation in distance seen between samples (PcoA loading axis 1), mostly due to dilution 3 samples. In line with this shift in distance, microbiota profiles of dilution 3 showed significant increase in relative abundance of Proteobacteria (Figure 2, ANOVA p<0.001) and significant decrease in Bacteroidetes (ANOVA p<0.001) compared to undiluted saliva. For all methods except Mobio, diversity increased in samples below 10^6^ bacteria per ml compared to undiluted saliva (Shannon diversity: mean 3.29 versus 2.79, (stdev 0.15 and 0.14), Simpson index: mean 0.08 versus 0.14 (stdev 0.02 and 0,007) for dilution 3 samples versus undiluted saliva respectively, table 1). This increase in diversity was mostly reflected in an increase in low abundant genera and unique sequences apparently not present in the original saliva sample. To test this, we combined all sequence data of the undiluted and dilution 1 and 2 samples (excluding Mobio dilution 1 and 2) considering a total of 61.057 raw sequences and concluded that in this large pool of sequences we did not observe the majority of low abundant genera found in the highly diluted samples suggesting an external source of origin. Most of these low abundant sequences corresponded to the taxon Proteobacteria.

### Effect of bacterial density in upper airway niches

To extrapolate the results of the salivary dilution experiments to human low-density niches we obtained samples from two low-density communities: nares and nasopharynx (0.1–10 pg/µl, table S1) of four healthy adult volunteers. For each sample DNA was extracted by four different methods, and the microbiota analysed by 16srDNA 454 pyrosequencing.

**Figure 3:**
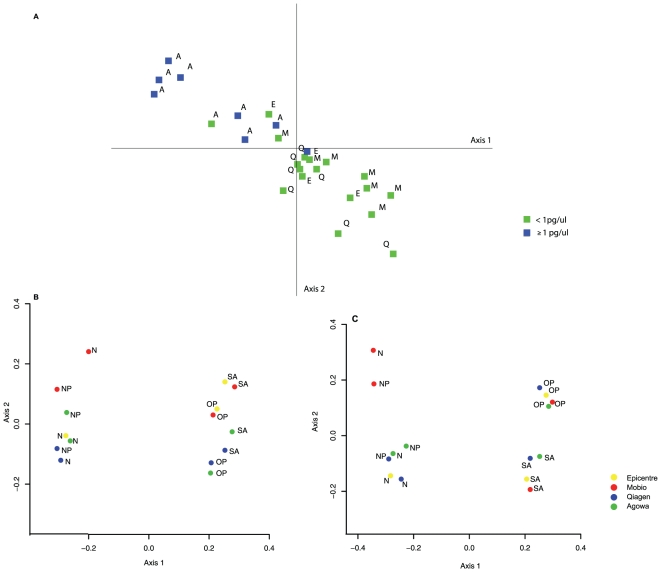
Principal component analyses (PCA) of the microbiota profiles and Principal Coordinate of Analyses (PcoA) plot of the weighted and unweighted UniFrac average distance per site and DNA extraction method. *a*. Principal component analyses (PCA) of the microbiota profiles of the nares and nasopharynx depicted per dilution and DNA extraction method. Depicted in colors are 16S DNA levels (blue = ≥1 pg/µl, green = <1 pg/µl). Depicted in characters are the DNA extraction methods (A = Agowa, E = Epicentre, Q = qiagen, M = Mobio). Clustering of the samples is according to DNA level and DNA extraction method. Differences in template concentration were due to differences in DNA extraction efficiency between used methods and effects of template concentration on microbiota analyses were therefore fully tied to DNA extraction effects. *b*.PcoA plot of the weighted UniFrac. Shown in colored circles are the DNA extraction methods (yellow = Epicentre, red = Mobio, blue = Qiagen and green = Agowa). The abbreviations represent the site of sampling (NP = nasopharynx, N = nares, OP = oropharynx, SA = saliva). Clear clustering per site of sampling was observed with saliva and oropharynx distant from nares and nasopharynx samples. For the oropharynx and saliva clusters significant sub-clustering per DNA extraction method was seen with clusters of Epicentre and Mobio, distant from Agowa and Qiagen clusters. DNA extraction method in these high density sites even introduced a larger distance in microbiota profile than origin of the sample (saliva or oropharynx). *c*. PCoA plot of the unweighted UniFrac as described above. Clear clustering per site of sampling was observed with saliva and oropharynx distant from nares and nasopharynx samples, and also between saliva and oropharynx. For the nares and nasopharynx clusters significant sub-clustering per DNA extraction method was seen with clusters of Agowa, Qiagen and Epicentre distant from Mobio. Both weighted and unweighted UniFrac analysis of sequence data revealed distinct clustering of saliva and oropharyngeal separate from nares and nasopharynx samples, reflecting unique differences in microbiota composition between these sites (Amova, p<0.001, Figure S4 and S5).

Analysis of the microbiota community structure of low-density nares and nasopharynx samples revealed apparent grouping of samples in which template concentration was less than 1 pg/µl and those in which template concentration was higher than 1 pg/µl (Figure 3a). In line with the saliva dilution experiments, significantly higher levels of Proteobacteria were found in the samples with less than 1 pg/µl compared to identical samples extracted with higher yields and at template levels above this threshold (ANOVA p<0.001, mean 22.2 and 11.09%, stdev 23.03 and 15.36% respectively). The relative abundance of Bacteroidetes was however not significantly reduced below this threshold as was found in the dilution experiment. It is important to note that the differences in template concentration were due to differences in DNA extraction efficiency between used methods. So, despite clear correlation between DNA level and microbiota analyses, effects were fully tied to differences in DNA extraction methodology.

### Effect of extraction methodology

To study the effect of DNA extraction method on microbiota analyses in a DNA recovery range that would not interfere profoundly with microbiota analyses, we sampled two high-density communities: oropharynx and the oral cavity (saliva, 10^3^–10^4^ pg/µl, table S1), besides two low-density communities: nares and nasopharynx. DNA was extracted by the same four methods and microbiota analysed by 454 pyrosequencing.

For these high density communities most profound differences between extraction methods were observed in relative abundance of taxa (weighted UniFrac, figure 3b and S4) and to a minor extent to taxa representation (unweighted UniFrac, figure 3c and S5), in which Agowa and Qiagen were similar (procrustes analyses, p<0.05) and both significantly distant from Mobio and Epicentre (Amova p<0.006).

**Figure 4:**
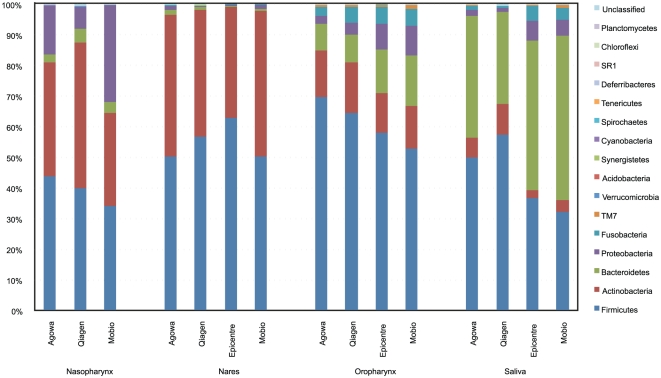
Phyla composition of individual 1 per sample site and DNA extraction method. Samples extracted with Agowa and Qiagen showed a significant higher proportion of Actinobacteria and Firmicutes and a lower proportion of Bacteriodetes, compared to Epicentre and Mobio, especially in the oropharyngeal and saliva samples for all 4 individuals. Note that nasopharyngeal samples from all four individuals isolated with Epicentre failed to give results.

Differences in microbiota profile were expressed by a significant higher proportion of Actinobacteria and Firmicutes and a lower proportion of Bacteriodetes in the Agowa and Qiagen method, compared to Epicentre and Mobio (Figure 4, significant p<0.05). The higher proportion of Actinobacteria obtained with Agowa and Qiagen was reflected by a significant higher abundance of the genera *Rothia* and *Actinomyces* and presence of several low abundant ones. In addition, Agowa and Qiagen yielded a higher abundance of the Firmicute *Streptococcus*, but lower abundance of Firmicutes *Veillonella* and *Staphylococcus*. In the Bacteroidetes phylum, lower abundance of *Porphyromonas* and *Prevotella* was observed in the Agowa and Qiagen extracted samples compared with Mobio and Epicentre (Figure 4 and S6).

We observed similar differences in the ability to capture DNA between methods in low-density samples nares and nasopharynx (Figure 4 and S6). Both variation in relative abundance as well as in species representation differed between methods, suggesting for the latter finding an intertwining effect of DNA recovery below 1 pg/µl. Overall bacterial DNA recovery varied 100 fold between methods (Table S1) with highest levels retrieved by the Agowa and Epicentre method. Both extraction methods retrieved DNA levels for these niches above 1 pg/µl, while Qiagen and Mobio retrieved DNA levels below 1 pg/µl.

## Discussion

Human habitats contain unique bacterial communities at different density depending on the habitat structure and community function. The upper airways are colonized with bacterial communities at low density, including the nasopharynx and nares, as was illustrated in the manuscript. These bacterial communities however contain potential respiratory pathogens, but also act as first-line defense against overgrowth and invasion of those pathogens leading to respiratory infections. To study these important microbiota, accurate processing for sequencing and potential limitations for working with these materials should be understood. In this study we show the effect of bacterial density on microbiota analyses by using serially diluted saliva samples and samples from low density (nares and nasopharynx) communities of the upper respiratory tract to identify potential limitations and biases on microbiota analyses.

By diluting a given sample, we expected low abundant genera to disappear first from the profiles as the effect of subsequent reduction in taxa richness, as described previously [10]. Interestingly however, for all dilutions, sequences affiliated to ‘low abundant’ genera (mostly Firmicutes) increased whereas those affiliated to ‘high abundant’ genera (mostly Bacteroidetes) decreased. This phenomenon might be explained by changes in 16S amplification efficiency due to minor differences in nucleotide composition of the primer sequence [13], [14], [15]. Highest melting temperature (Tm), presumably positively affecting hybridization efficiency, was found for both forward (+2°C) and reverse (+0.8°C) primer sequences of the Firmicute *Veillonella* compared to both *Prevotella* (decreased in abundance) and *Streptococcae* (sustained in abundance). This could potentially explain the increase in abundance found for this genus in diluted samples, though not for other genera. Therefore, additional mechanisms must contribute to this phenomenon. Despite these differences in abundance, bacterial representation in the undiluted saliva sample and diluted samples up to a density of 10^7^ to 10^6^ bacteria per ml was highly in concordance.

In contrast, at a density of <10^6^ bacteria per ml and DNA <1 pg/µl of template we observed clear shifts in microbiota profiles for all DNA extraction methods. Not only relative abundance of bacteria changed, but also bacterial representation was affected by diluting, with an apparent loss of certain bacterial species present in the original sample. Moreover, detection of (low abundant) species, mostly proteobacteria, not present in the original samples was observed. To be able to exclude a lack of sensitivity in analyses of the undiluted samples with respect to those newly observed in diluted samples, we pooled all sequences of the undiluted and diluted samples with densities of 10^7^ to 10^6^ bacteria per ml (61.057 sequences) and still did not observe most of the genera that increased at this density. The fact, however, that a similar increase in OTUs was observed in the (undiluted) samples <1 pg/µl from both the nares and nasopharynx but not the ones above this threshold strongly supports the validity of this finding. Although we can't rule out that these low abundant OTUs in samples <1 pg/µl could still be a true reflection of diversity of the microbiome, more likely background contamination from chemicals and solutions used prior to amplicon preparation could have introduced these genera, which is in line with previous reports [16], [17], [18]. Also in line with our results, Tanner et al. found that most of the contaminant rDNA sequences from negative extraction controls belonged to the phylum Proteobacteria. Furthermore he reports that many of the clones they obtained from the negative extraction controls were closely related to sequences recovered from environmental samples [18]. Many of the betaproteobacteria are found in wastewater and soil environments and can therefore easily contaminate chemicals and solutions used for DNA extraction. In especially low-density samples, DNA levels are not high enough to overrule contaminant DNA in the analyses and may therefore cause the observed shifts in microbiota profiles in samples below 10^6^ bacteria per ml. We therefore recommend based on these results a lower bacterial density threshold of 10^6^ bacteria per ml and template DNA of 1 pg/µl.

In this study we didn't test for methods to reduce background DNA. Development of effective and efficient decontamination methods also suitable for high-throughput use or development of ultrapure reagents could potentially further reduce background DNA [16], [19].

Furthermore, this study was performed on samples from different niches of the upper respiratory and oral tract. Therefore, applying our findings to other niches should be approached with caution. However, taking into account the clear shifts at the density level at 10^5^ bacteria per ml and DNA <1 pg/µl of template, we expect these shifts will also occur in other types of communities depending on density.

In low density samples, DNA extraction method determined if DNA levels of similar samples were below or above the DNA template threshold of 1 pg/µl. The ability to capture DNA efficiently in these samples is therefore of importance. In that context we studied what extraction method was best in both efficient and representative DNA recovery. Considering that at low density, effects of lysis preference per method intertwined with low DNA template-induced effects, we studied differences between extraction methods first in high density regions: saliva and oropharynx. DNA extraction method in these regions mostly influenced the relative abundance of bacteria and these findings are in line with the DNA extraction effects previously observed on fecal microbiota structures [5], [9]. The observed differences between methods might be explained by the variation in lyses preferences between methods as gram-positive bacteria in the phyla *Actinobacteria* and *Firmicutes*, are harder to lyse with enzymatic methods because of their ‘tough’ cell walls. DNA extraction utilizing a mechanical approach seemed to be giving higher representation of these hard to lyse and highest overall 16S DNA levels. This is in agreement with literature, where also mechanical lysis with bead beating in combination with phenol was found to perform best in extracting representative DNA from faecal material [5], [9], [11], [20]. In low density samples, Agowa retrieved highest DNA levels and considering both this and the ability to capture all taxa including the hard to lyse taxa, Agowa extraction method seemed to perform best for low density samples.

In summary, we show a clear boundary of accurate microbiota profiling of density level of <10^6^ bacteria per ml and DNA concentration below 1 pg/µl. Below this density level, microbiota profiles seem to become distinct from samples of higher density potentially introducing a serious error in both species representation as well as abundance. Similar samples can vary in retrieved DNA levels by 10–100 fold depending on DNA extraction methodology and can influence microbiota analyses to a greater extent in low density regions.

## Materials and Methods

### Sample collection and storage

We sampled nasopharynx, nares, oropharynx and saliva of 4 healthy adult volunteers. Exclusion criteria were symptoms of a respiratory infection or prior antibiotic use. Samples were taken at least two hours before or after brushing teeth. Written informed consent was obtained from the participants. This study received approval by the ethics committee of the University Medical Center Utrecht. The study was performed in accordance with the European Statements for Good Clinical Practice, which includes the provisions of the Declaration of Helsinki.

Nasopharynx, nares and oropharynx samples were collected using Copan eswabs and stored in 1 ml liquid Amies Medium (483CE, Copan Diagnostics Inc. , CA). Saliva was collected in the morning before breakfast was consumed by spitting in a 15 ml Falcon tube. Samples were aliquoted per 100 µl in 1.5 ml screw-cap eppendorf tubes (Sarstedt, Nümbrecht, Germany) and stored at −80°C until further analysis. Tenfold dilutions of saline down to 10^−5^ were made from the original undiluted saliva sample of individual 1 and in 1 ml aliquots stored at −70°C.

### DNA extraction protocols

All DNA extractions were performed with 100 µl of the original sample. Based on literature [1], [5], [9], [11], [21], [22], four DNA extraction methods were tested: Epicentre Masterpure ™ DNA purification kit (catalogue MCD85201, Epicentre technologies, Madison), Qiagen DNeasy Blood & Tissue Kit (Catalogue 69504, Qiagen, Hilden, Germany), MoBio PowerSoil DNA Extraction Kit (Catalogue 12888-05, Mo Bio Laboratories, Carlsbad, CA, USA) and phenol/bead beating in combination with the Agowa Mag mini DNA extraction kit (Catalogue 40401, LGC Genomics, Berlin, Germany). To increase DNA yields, DNA extracted with all methods was eluted with relatively small volume of 50 µl of recommended elution buffer.

For the Epicentre Masterpure DNA purification kit (Epicentre) DNA was isolated exactly as per the manufacturers instructions. This method is based on enzymatic/chemical and thermal lysis of cells and DNA precipitation with isopropanol (2-propanol, baker analyzed ACS reagent, catalogue, 9084-03, Avantor performance materials, Phillipsburg NJ).

Qiagen DNeasy Blood & Tissue kit (Qiagen) is also based on enzymatic and chemical lysis with longer incubation at 37 and 56 degrees Celsius. The enzymatic lysis buffer per sample consisted of 0.02 M TrisHCl pH 8, 0. 2 mM Sodium EDTA, 1.2% triton X-100 (catalogue T1503-1kg, catalogue ED2SS-1KG, catalogue 9002-93-1 respectively, Sigma, Saint Louis, MO), 20 mg/µl lysozyme (Kit content) as described in the manufacturers protocol. We added 200 units of mutanolysine (catalogue M9901,Sigma, MS) to this lysis buffer to further facilite bacterial cell lysis. The purification is performed by column binding as per manufacturers instruction.

Mobio PowerSoil DNA Extraction Kit (Mobio) is a DNA extraction procedure based on mechanical and chemical lysis. DNA was isolated exactly as stated in manufracteres' instruction.

The last method tested was the phenol/bead beating in combination with Agowa Mag mini DNA extraction kit (Catalogue40401, LGC genomics, Berlin, Germany). Per 0.1 ml sample 0.3 g zirconium beads (diameter 0,1 mm, catalogue 11079101z, Biospec Products, Bartlesville, OK), 0,25 ml lysis buffer (Agowa Mag mini DNA extraction kit and 0.2 ml phenol (Phenol solution BioUltra, catalogue P4557, Sigma-Aldrich, St Louis, MO) was added. The sample was mechanically disrupted by bead beating 2 times for 3 min (Mini-Beadbeater 16, catalogue 607EUR, Biospec Products, Bartlesville, OK. The homogenate was placed on ice for at least 1 minute after each beating cycle and centrifuged at 1800 rcf (4000 rpm) for 10 minutes to separate the aqueous and phenolic phases. The aqueous phase was transferred to a new screw-cap Eppendorf tube and binding buffer and magnetic beads, twice the volume of supernatant, were added. To maximize recovery, we incubated samples for 30 minutes instead of 10 min recommended by kit's manufacturer. The remaining steps were executed according to the Agowa Mag Mini DNA extraction protocol.

### Real time PCR for bacterial DNA

Total bacterial load was analyzed by quantitative PCR (7500 Fast Real-Time PCR system, Applied Biosystems, catalogue 4351107, Foster City, CA) using universal primers-probe set targeting the 16S rDNA gene, as described previously [6]. All samples were processed in quadruplicates.

CT values were related to the standard curve ranging from 0.1 pg/µl to 1 ng/µl of bacterial DNA. The reference DNA for the q-PCR standard curve was purified from 800 µl human saliva spiked with 10^4^-bacteria of 6 orals strains, *Streptococcus mutans, Fusobacterium nucleatum, Porphyromonas gingivalis, Porphyromonas catoniae, Propionibacterium propionicum, Tannerella forsythia*. Total bacterial DNA was quantified by nanodrop and diluted to fit targeted concentrations. The reported qPCR results adhere to the MIQE standards for reporting qPCR data (Methods S2).

### Amplicon library preparation

Generation of PCR amplicon library was performed by amplification of the small subunit ribosomal RNA gene V5–V7 hypervariable region as described previously [6]. In short, samples with DNA recovery of equal or less then 10 pg/µl of DNA were cycled 35 times instead of 30 times for adequate amplicon recovery using the same protocol. Amplicons were size checked, quantified and equimolar pooled and purified from agarose gel. The library was sequenced in the 454 GS-FLX-Titanium Sequencer (Life Sciences (Roche), Branford, CT).

### Melting temperature calculation for strains from the o.m.d

To compare primer sequences for G/C content and primer mismatch we used reference genomes from the human oral microbiota database (http://www.homd.org/, retrieved at July 18^th^ 2011) and calculated primer mismatches per primer sequence and melting temperature (Tm) for each strain by the formula Tm = 64.9+41*(yG+zC-16.4)/(wA+xT+yG+zC) [23].

### Sequence processing and analyses

FASTA-formatted sequences and corresponding quality scores were extracted from the .sff data file generated by the GS-FLX-Titatium sequencer using the GS Amplicon software package (Roche, Branford, CT) and processed using modules implemented in the Mothur v. 1.20.0. software platform according to previously described methods **(** Methods S1) [24]. On average 7000 raw sequences were generated per sample. Five samples failed to give results (table S2). Sequences were de-noised using a pseudo-single linkage algorithm with the goal of removing sequences that are likely due to pyrosequencing errors (“pre.cluster” command) [25]. A total of 11814 potentially chimeric sequences were detected and removed using the “chimera.slayer” command [26]. High quality aligned sequences were classified using the RDP-II naïve Bayesian Classifier[27]. Aligned sequences were clustered into OTUs (defined by 97% similarity) using the average linkage clustering method. Sequences were normalized to 1000 sequences per samples. Five additional samples were removed from the analyses because of low sequence number. For each of the remaining samples rarefaction curves were plotted and community diversity parameters (Shannon diversity index, Chao1 and Simpson's) calculated. Detailed information on sequence characteristics and the number of assigned sequences per tax-level can be found in the supplemental material (Table S2 and S3).

Sequence data were, amongst others, subjected to weighted and unweighted UniFrac analysis using the UniFrac module implemented in Mothur [28]. The UniFrac metric is a proxy for the distance between different microbial communities taking into account the phylogenetic relatedness of lineages in each sample. The unweighted algorithm performs the analysis based on presence or absence of bacterial lineages, whereas weighted UniFrac also accounts for relative sequence abundance. The phylogenetic dendrogram for the UniFrac analyses was obtained by FastTree [29]. Clustering was visualized using Principal Coordinates of Analyses for the unweighted UniFrac of the serially diluted saliva and for the calculated average UniFrac distances per niche and extraction method. UniFrac dendograms were displayed using iTOL: interactive Tree of Life [30]. We used the distance-based AMOVA in the Mothur pipeline to assess significant differences between branches, and the Procrustes analyses from the Protest package in R to calculate significant similarities. To test the stability of the UniFrac dendogram we calculated Jackknife support values in R (http://onlinelibrary.wiley.com/doi/10.1111/j.1467-9868.2005.00489.x/abstract) and performed random permutations (1000) in Mothur. Microbiota composition column graphs were drawn in excel. Unsupervised data-analyses, hierarchical clustering, Principal Component Analyses and Significant Analyses of Microarrays (SAM) analyses were performed in the MeV software package as part of TM4 microarray software suite [31]. To obtain significant differences between microbiota profiles, we used the Pearson's correlation with average linkage clustering method and FDR significance. In these latter analyses, OTUs represented by <3 sequences were removed from the analyses.

## Supporting Information

Figure S1 **16S DNA levels of 154 nasopharyngeal swabs of infants** [**6**] **.** After DNA extraction 16S template quantity was quantified by universal 16S qPCR. DNA quantity scattered from <0.5 pg/µl to o.12 ng/µl with an average of 7.4 pg/µl.
(EPS)

Figure S2 **Weighted UniFrac dendogram of all samples depicted according to dilution (undiluted saliva, dilution 1,2 and 3 ,representing 10^7^ to 10^5^ bacteria per ml, respectively) and DNA extraction method.** Shown in colored circles are the DNA extraction methods (yellow = Epicentre, red = Mobio, blue = Qiagen and green = Agowa). The abbreviations represent the dilutions (1 = dilution 1 (10^7^ bacteria per ml), 2 = dilution 2 (10^6^ bacteria per ml) and 3 = dilution 3 (10^5^ bacteria per ml)). The last column depicts the Jackknife support values per sample for creating 7 clusters (threshold is shown as a dashed line).
(EPS)

Figure S3 **Unweighted UniFrac dendogram of all samples depicted according to dilution (undiluted saliva, dilution 1,2 and 3 ,10^7^ to 10^5^ bacteria per ml, respectively) and DNA extraction method.** Shown in colored circles are the DNA extraction methods (yellow = Epicentre, red = Mobio, blue = Qiagen and green = Agowa). The abbreviations represent the dilutions (1 = dilution 1 (10^7^ bacteria per ml), 2 = dilution 2 (10^6^ bacteria per ml) and 3 = dilution 3 (10^5^ bacteria per ml)). The last column depicts the Jackknife support values per sample for creating 7 clusters (threshold is shown as a dashed line). Great overlap in sequence representation was seen between undiluted samples and samples diluted up to dilution 2 (10^6^ bacteria per ml) for all DNA extraction methods except for Mobio. Dilution 3 samples were significant distant from the other dilutions.
(EPS)

Figure S4 **Weighted UniFrac dendogram of all samples depicted according to site, DNA extraction method and individual.** The first column represent the site of sampling (NP = nasopharynx, N = nares, OP = oropharynx). Shown in colored circles are the DNA extraction methods (yellow = Epicentre, red = Mobio, blue = Qiagen and green = Agowa). Participating individuals were numbered and the third column depicts the particular individual the sample was collected from. The last column represents the Jackknife support values per sample for creating 19 clusters (threshold is shown as a dashed line). Clear clustering per site of sampling was observed. For the oropharynx and saliva clusters significant sub-clustering per DNA extraction method was seen with clusters of Epicentre and Mobio, distant from Agowa and Qiagen clusters.
(EPS)

Figure S5 **Unweighted UniFrac dendogram of all samples depicted according to site, DNA extraction method and individuals.** The first column represent the site of sampling (NP = nasopharynx, N = nares, OP = oropharynx). Shown in colored circles are the DNA extraction methods (yellow = Epicentre, red = Mobio, blue = Qiagen and green = Agowa). Participating individuals were numbered and the third column depicts the particular individual the sample was collected from. The last column represents the Jackknife support values per sample for creating 21 clusters (threshold is shown as a dashed line). Clear clustering per site of sampling was observed. For the nares and nasopharynx clusters significant sub-clustering per DNA extraction method was seen with clusters of Epicentre and Mobio, distant from Agowa and Qiagen. Agowa and Qiagen were, however, also significantly distant from each other.
(EPS)

Figure S6 **Heatmap and weighted UniFrac dendrogram of saliva and nares samples of individual 1 as obtained by the 4 extraction methods.** Phyla and genera found at these sites are depicted. Number and color depict the relative abundance percentage per genus in the heatmap. Clear variation in profiles between the different DNA extraction methods was seen, with more abundant genera belonging to the phyla Actinobacteria and Firmicutes in the Agowa and Qiagen isolated samples. In the nares samples more variation in presence or absence of genera was seen between DNA extraction methods as compared to saliva. Significant Analyses of Microarrays (SAM) analyses were performed in the MeV software package as part of TM4 microarray software suite and significant genes are marked with asterixes.
(EPS)

Table S1 **Recovery of DNA, representing the mean of quadruplicate measurements, is shown per site, DNA isolation method and individual.** DNA quantity is measured by q-PCR using universal primers-probe set targeting the 16S rDNA gene and depicted in picogram per µl. Highest DNA yields were obtained using the Agowa method, followed by the Epicentre extraction method. Agowa = Bead beating, phenol and Agowa Mag mini DNA isolation kit. Epicentre = Epicentre Masterpure DNA Purification Kit, Qiagen = Qiagen Dneasy Blood & Tissue kit . Mobio = Mobio Powersoil DNA isolation kit.
(DOC)

Table S2 **Overview of 16S DNA levels, achieved number of reads, the number of retained reads after filtering and the number of unique sequences per sample.** Furthermore, diversity indices, the coverage and number of estimated taxa and genera per samples are provided.
(DOC)

Table S3 **Number of sequences that were able to be classified per taxonomic level.**
(DOC)

Methods S1 **More detailed information on sequence preprocessing and filter regimen.**
(DOC)

Methods S2 **MIQE form: Reporting requirement for Quantitative PCR Assays.**
(DOC)

## References

[1] Costello EK, Lauber CL, Hamady M, Fierer N, Gordon JI (2009). Bacterial community variation in human body habitats across space and time.. Science.

[2] Dethlefsen L, McFall-Ngai M, Relman DA (2007). An ecological and evolutionary perspective on human-microbe mutualism and disease.. Nature.

[3] Peterson J, Garges S, Giovanni M, McInnes P, Wang L (2009). The NIH Human Microbiome Project.. Genome Res.

[4] Spor A, Koren O, Ley R (2011). Unravelling the effects of the environment and host genotype on the gut microbiome.. Nat Rev Microbiol.

[5] Salonen A, Nikkila J, Jalanka-Tuovinen J, Immonen O, Rajilic-Stojanovic M (2010). Comparative analysis of fecal DNA extraction methods with phylogenetic microarray: effective recovery of bacterial and archaeal DNA using mechanical cell lysis.. J Microbiol Methods.

[6] Bogaert D, Keijser B, Huse S, Rossen J, Veenhoven R (2011). Variability and diversity of nasopharyngeal microbiota in children: a metagenomic analysis.. PLoS One.

[7] Parker CT, Sperandio V (2009). Cell-to-cell signalling during pathogenesis.. Cellular microbiology.

[8] Boyer M, Wisniewski-Dye F (2009). Cell-cell signalling in bacteria: not simply a matter of quorum.. FEMS microbiology ecology.

[9] Wu GD, Lewis JD, Hoffmann C, Chen YY, Knight R (2010). Sampling and pyrosequencing methods for characterizing bacterial communities in the human gut using 16S sequence tags.. BMC Microbiol.

[10] Wu JY, Jiang XT, Jiang YX, Lu SY, Zou F (2010). Effects of polymerase, template dilution and cycle number on PCR based 16 S rRNA diversity analysis using the deep sequencing method.. BMC Microbiol.

[11] Zoetendal EG, Heilig HG, Klaassens ES, Booijink CC, Kleerebezem M (2006). Isolation of DNA from bacterial samples of the human gastrointestinal tract.. Nat Protoc.

[12] van Gils EJ, Veenhoven RH, Hak E, Rodenburg GD, Bogaert D (2009). Effect of reduced-dose schedules with 7-valent pneumococcal conjugate vaccine on nasopharyngeal pneumococcal carriage in children: a randomized controlled trial.. JAMA : the journal of the American Medical Association.

[13] Hayward AL, Oefner PJ, Sabatini S, Kainer DB, Hinojos CA (1998). Modeling and analysis of competitive RT-PCR.. Nucleic Acids Res.

[14] Polz MF, Cavanaugh CM (1998). Bias in template-to-product ratios in multitemplate PCR.. Appl Environ Microbiol.

[15] Sipos R, Szekely AJ, Palatinszky M, Revesz S, Marialigeti K (2007). Effect of primer mismatch, annealing temperature and PCR cycle number on 16S rRNA gene-targetting bacterial community analysis.. FEMS Microbiol Ecol.

[16] Corless CE, Guiver M, Borrow R, Edwards-Jones V, Kaczmarski EB (2000). Contamination and sensitivity issues with a real-time universal 16S rRNA PCR.. Journal of clinical microbiology.

[17] Muhl H, Kochem AJ, Disque C, Sakka SG (2010). Activity and DNA contamination of commercial polymerase chain reaction reagents for the universal 16S rDNA real-time polymerase chain reaction detection of bacterial pathogens in blood.. Diagnostic microbiology and infectious disease.

[18] Tanner MA, Goebel BM, Dojka MA, Pace NR (1998). Specific ribosomal DNA sequences from diverse environmental settings correlate with experimental contaminants.. Applied and environmental microbiology.

[19] Klaschik S, Lehmann LE, Raadts A, Hoeft A, Stuber F (2002). Comparison of different decontamination methods for reagents to detect low concentrations of bacterial 16S DNA by real-time-PCR.. Mol Biotechnol.

[20] Zoetendal EG, Ben-Amor K, Akkermans AD, Abee T, de Vos WM (2001). DNA isolation protocols affect the detection limit of PCR approaches of bacteria in samples from the human gastrointestinal tract.. Syst Appl Microbiol.

[21] Sommer MO, Dantas G, Church GM (2009). Functional characterization of the antibiotic resistance reservoir in the human microflora.. Science.

[22] Gao Z, Tseng CH, Pei Z, Blaser MJ (2007). Molecular analysis of human forearm superficial skin bacterial biota.. Proc Natl Acad Sci U S A.

[23] Chen T, Yu WH, Izard J, Baranova OV, Lakshmanan A (2010). The Human Oral Microbiome Database: a web accessible resource for investigating oral microbe taxonomic and genomic information.

[24] Schloss PD, Westcott SL, Ryabin T, Hall JR, Hartmann M (2009). Introducing mothur: open-source, platform-independent, community-supported software for describing and comparing microbial communities.. Appl Environ Microbiol.

[25] Huse SM, Welch DM, Morrison HG, Sogin ML (2010). Ironing out the wrinkles in the rare biosphere through improved OTU clustering.. Environ Microbiol.

[26] Haas BJ, Gevers D, Earl AM, Feldgarden M, Ward DV (2011). Chimeric 16S rRNA sequence formation and detection in Sanger and 454-pyrosequenced PCR amplicons.. Genome Res.

[27] Cole JR, Chai B, Marsh TL, Farris RJ, Wang Q (2003). The Ribosomal Database Project (RDP-II): previewing a new autoaligner that allows regular updates and the new prokaryotic taxonomy.. Nucleic acids research.

[28] Lozupone C, Knight R (2005). UniFrac: a new phylogenetic method for comparing microbial communities.. Appl Environ Microbiol.

[29] Price MN, Dehal PS, Arkin AP (2009). FastTree: computing large minimum evolution trees with profiles instead of a distance matrix.. Molecular biology and evolution.

[30] Letunic I, Bork P (2007). Interactive Tree Of Life (iTOL): an online tool for phylogenetic tree display and annotation.. Bioinformatics.

[31] Saeed AI, Bhagabati NK, Braisted JC, Liang W, Sharov V (2006). TM4 microarray software suite.. Methods Enzymol.

