# Supplementary material for: Deep Sequencing Analyses of Low Density Microbial Communities: Working at the Boundary of Accurate Microbiota Detection
Source: PLoS One. 2012 Mar 6;7(3):e32942. doi: 10.1371/journal.pone.0032942 (PMC3295791; doi:10.1371/journal.pone.0032942)
Supplement: Table S1 — Recovery of DNA, representing the mean of quadruplicate measurements, is shown per site, DNA isolation method and individual. DNA quantity is measured by q-PCR using universal primers-probe set targeting the 16S rDNA gene and depicted in picogram per µl. Highest DNA yields were obtained using the Agowa method, followed by the Epicentre extraction method. Agowa = Bead beating, phenol and Agowa Mag mini DNA isolation kit. Epicentre = Epicentre Masterpure DNA Purification Kit, Qiagen = Qiagen Dneasy Blood & Tissue kit . Mobio = Mobio Powersoil DNA isolation kit. (DOC) [file pone.0032942.s007.doc]

**Table S1** Recovery of DNA, representing the mean of quadruplicate measurements, is shown per site, DNA isolation method and individual.

| **DNA isolation method** | | **Agowa** | **Epicentre** | **Mobio** | **Qiagen** |
| --- | --- | --- | --- | --- | --- |
| **Saliva** | Individual 1 | 8,39E+03 | 5,83E+03 | 7,90E+02 | 1,03E+03 |
|  | Individual 2 | 8,76E+03 | 4,90E+03 | 1,44E+03 | 1,47E+02 |
|  | Individual 3 | 9,85E+03 | 5,61E+03 | 8,76E+02 | 8,53E+02 |
|  | Individual 4 | 6,62E+03 | 3,57E+03 | 7,17E+02 | 1,22E+03 |
| **Oropharynx** | individual 1 | 5,48E+02 | 2,12E+02 | 5,33 | 5,47 |
|  | Individual 2 | 5,75E+02 | 2,81E+02 | 1,25E+01 | 2,52 |
|  | Individual 3 | 4,00E+02 | 5,84E+01 | 8,20E+0 | 4,38E+01 |
|  | Individual 4 | 9,01E+02 | 4,95E+02 | 1,26E+01 | 8,54 |
| **Nasopharynx** | Individual 1 | 1,58 | 1,64 | 1,65E-01 | 2,05E-01 |
|  | Individual 2 | 8,48E-01 | 8,71E-01 | 1,67E-01 | 9,60E-01 |
|  | Individual 3 | 3,80 | 3,85 | 1,85^E^-01 | 5,12E-01 |
|  | Individual 4 | 1,75 | 1,72 | 1,28E-01 | 2,22E-01 |
| **Nares** | Individual 1 | 3,49E+01 | 1,45E+01 | 6,52E-01 | 3,00E-01 |
|  | Individual 2 | 1,62 | 7,99E-01 | 1,71E-01 | 7,88E-02 |
|  | Individual 3 | 5,81 | 9,01E-01 | 5,77E-01 | 1,04E-01 |
|  | Individual 4 | 3,92 | 4,73E-01 | 1,69E-01 | 7,60E-02 |

DNA quantity is measured by q-PCR using universal primers-probe set targeting the 16S rDNA gene and depicted in picogram per µl. Highest DNA yields were obtained using the Agowa method, followed by the Epicentre extraction method. Agowa =Bead beating, phenol and Agowa Mag mini DNA isolation kit. Epicentre = Epicentre Masterpure DNA Purification Kit, Qiagen = Qiagen Dneasy Blood & Tissue kit . Mobio = Mobio Powersoil DNA isolation kit.
