# Supplementary material for: Deep Sequencing Analyses of Low Density Microbial Communities: Working at the Boundary of Accurate Microbiota Detection
Source: PLoS One. 2012 Mar 6;7(3):e32942. doi: 10.1371/journal.pone.0032942 (PMC3295791; doi:10.1371/journal.pone.0032942)
Supplement: Table S2 — Overview of 16S DNA levels, achieved number of reads, the number of retained reads after filtering and the number of unique sequences per sample. Furthermore, diversity indices, the coverage and number of estimated taxa and genera per samples are provided. (DOC) [file pone.0032942.s008.doc]

| **Samples** | **DNA isolation method** | **Niche** | **qPCR (pg/ul)** | **Person** | **# reads/**  **sample** | **# maintained reads after filtering** | **# Unique sequences** | **# sequences after normalization** | **# Unique sequences** | **coverage** | **Npshannon**  **Diversity**  **index** | **Simpson**  **Diversity**  **index** | **#Taxa** | **# genera** |
| --- | --- | --- | --- | --- | --- | --- | --- | --- | --- | --- | --- | --- | --- | --- |
| **1** | Agowa | Nasopharynx | 1.58E+00 | 1 | 6150 | 2231 | 2165 | 1000 | 108 | 0.943 | 3.365685 | 0.076589 | 9 | 92 |
| **2** | Agowa | Nasopharynx | 8.48E-01 | 2 | 4789 | 1108 | 1067 | 1000 | 97 | 0.968 | 3.346195 | 0.084599 | 9 | 65 |
| **3** | Agowa | Nasopharynx | 3.80E+00 | 3 | 7094 | 3398 | 3382 | 1000 | 53 | 0.979 | 1.62911 | 0.364581 | 7 | 64 |
| **4** | Agowa | Nasopharynx | 1.75E+00 | 4 | 6507 | 2209 | 2153 | 1000 | 115 | 0.956 | 3.128747 | 0.139115 | 8 | 86 |
| **5** | Agowa | Nares | 3.49E+01 | 1 | 14909 | 5254 | 5188 | 1000 | 75 | 0.964 | 2.766383 | 0.123658 | 9 | 70 |
| **6** | Agowa | Nares | 1.62E+00 | 2 | 10932 | 2789 | 2708 | 1000 | 126 | 0.948 | 3.563679 | 0.082621 | 9 | 105 |
| **7** | Agowa | Nares | 5.81E+00 | 3 | 19755 | 8689 | 8643 | 1000 | 56 | 0.975 | 1.672058 | 0.329566 | 8 | 84 |
| **8** | Agowa | Nares | 3.92E+00 | 4 | 10882 | 3533 | 3466 | 1000 | 125 | 0.938 | 3.387713 | 0.095938 | 10 | 105 |
| **9** | Agowa | Oropharynx | 5.48E+02 | 1 | 3408 | 1397 | 1383 | 1000 | 63 | 0.973 | 2.478799 | 0.212148 | 7 | 29 |
| **10** | Agowa | Oropharynx | 5.75E+02 | 2 | 4619 | 1986 | 1966 | 1000 | 53 | 0.977 | 2.348305 | 0.174346 | 7 | 26 |
| **11** | Agowa | Oropharynx | 4.00E+02 | 3 | 7674 | 2602 | 2529 | 1000 | 95 | 0.952 | 2.653243 | 0.187944 | 7 | 39 |
| **12** | Agowa | Oropharynx | 9.01E+02 | 4 | 5409 | 2017 | 1993 | 1000 | 52 | 0.978 | 1.963486 | 0.305443 | 7 | 30 |
| **13** | Agowa | Saliva dilution 1 | 6.12E+01 | 1 | 5719 | 2306 | 2306 | 1000 | 67 | 0.978 | 2.71213 | 0.1382 | 7 | 44 |
| **14** | Agowa | Saliva dilution 2 | 9.75E+00 | 1 | 4744 | 2119 | 2119 | 1000 | 75 | 0.97 | 3.069034 | 0.088603 | 6 | 52 |
| **15** | Agowa | Saliva dilution 3 | 8.42E-01 | 1 | 11871 | 4249 | 4249 | 1000 | 112 | 0.968 | 3.453665 | 0.074264 | 8 | 76 |
| **16** | Agowa | Saliva | 8.39E+03 | 1 | 4328 | 1668 | 1640 | 1000 | 90 | 0.951 | 2.720015 | 0.160146 | 7 | 38 |
| **17** | Agowa | Saliva | 8.76E+03 | 2 | 5063 | 2049 | 2001 | 1000 | 107 | 0.955 | 2.768648 | 0.147461 | 7 | 46 |
| **18** | Agowa | Saliva | 9.85E+03 | 3 | 3478 | 1313 | 1278 | 1000 | 94 | 0.94 | 2.904053 | 0.135409 | 7 | 35 |
| **19** | Agowa | Saliva | 6.62E+03 | 4 | 7239 | 3107 | 3081 | 1000 | 53 | 0.977 | 2.072568 | 0.251097 | 7 | 34 |
| **20** | Epicentre | Nasopharynx | 1.64E+00 | 1 | - | - | - | - | - | - | - | - | - | - |
| **21** | Epicentre | Nasopharynx | 8.71E-01 | 2 | - | - | - | - | - | - | - | - | - | - |
| **22** | Epicentre | Nasopharynx | 3.85E+00 | 3 | - | - | - | - | - | - | - | - | - | - |
| **23** | Epicentre | Nasopharynx | 1.72E+00 | 4 | - | - | - | - | - | - | - | - | - | - |
| **24** | Epicentre | Nares | 1.45E+01 | 1 | 10447 | 4093 | 4080 | 1000 | 38 | 0.984 | 2.30129 | 0.140344 | 7 | 39 |
| **25** | Epicentre | Nares | 7.99E-01 | 2 | 5713 | 1932 | 1913 | 1000 | 52 | 0.987 | 2.291361 | 0.285195 | 6 | 44 |
| **26** | Epicentre | Nares | 9.01E-01 | 3 | 13940 | 6159 | 6145 | 1000 | 30 | 0.981 | 1.502221 | 0.363077 | 5 | 34 |
| **Samples** | **DNA isolation method** | **Niche** | **qPCR (pg/ul)** | **Person** | **# reads/**  **sample** | **# maintained reads after filtering** | **# Unique sequences** | **# sequences after normalization** | **# Unique sequences** | **coverage** | **Npshannon**  **Diversity**  **index** | **Simpson**  **Diversity**  **index** | **#Taxa** | **# genera** |
| **27** | Epicentre | Nares | 4.73E-01 | 4 | 8185 | 2973 | 2963 | 1000 | 74 | 0.981 | 3.339871 | 0.067121 | 9 | 58 |
| **28** | Epicentre | Oropharynx | 2.12E+02 | 1 | 5857 | 2458 | 2438 | 1000 | 72 | 0.975 | 3.018685 | 0.08757 | 8 | 43 |
| **29** | Epicentre | Oropharynx | 2.81E+02 | 2 | 6457 | 2751 | 2732 | 1000 | 46 | 0.98 | 2.496328 | 0.128735 | 7 | 33 |
| **30** | Epicentre | Oropharynx | 5.84E+01 | 3 | 5037 | 2069 | 2043 | 1000 | 76 | 0.973 | 2.787305 | 0.127626 | 7 | 34 |
| **31** | Epicentre | Oropharynx | 4.95E+02 | 4 | 4790 | 2027 | 2003 | 1000 | 65 | 0.978 | 2.496222 | 0.142294 | 7 | 33 |
| **32** | Epicentre | Saliva dilution 1 | 7.84E+01 | 1 | 6768 | 2614 | 2614 | 1000 | 69 | 0.987 | 2.936752 | 0.111325 | 6 | 42 |
| **33** | Epicentre | Saliva dilution 2 | 3.34E+00 | 1 | 7353 | 3013 | 3013 | 1000 | 89 | 0.967 | 2.564074 | 0.242905 | 7 | 55 |
| **34** | Epicentre | Saliva dilution 3 | 1.77E+00 | 1 | 8635 | 3438 | 3438 | 1000 | 74 | 0.983 | 3.267258 | 0.081005 | 7 | 57 |
| **35** | Epicentre | Saliva | 5.83E+03 | 1 | 6394 | 2117 | 2096 | 1000 | 87 | 0.967 | 2.928939 | 0.14609 | 8 | 42 |
| **36** | Epicentre | Saliva | 4.90E+03 | 2 | 3477 | 1158 | 1130 | 1000 | 102 | 0.952 | 2.931348 | 0.129321 | 7 | 32 |
| **37** | Epicentre | Saliva | 5.61E+03 | 3 | 4535 | 1502 | 1451 | 1000 | 115 | 0.947 | 3.092533 | 0.135852 | 8 | 47 |
| **38** | Epicentre | Saliva | 3.57E+03 | 4 | 5404 | 1860 | 1827 | 1000 | 81 | 0.966 | 2.304411 | 0.217239 | 6 | 39 |
| **39** | Mobio | Nasopharynx | 1.65E-01 | 1 | 5398 | 1903 | 1898 | 1000 | 42 | 0.998 | 3.120661 | 0.059526 | 6 | 35 |
| **40** | Mobio | Nasopharynx | 1.67E-01 | 2 | 1008 | 278 | 275 | - | - | - | - | - | 5 | 23 |
| **41** | Mobio | Nasopharynx | 1.85E-01 | 3 | 8817 | 3471 | 3469 | 1000 | 32 | 0.994 | 1.819607 | 0.290132 | 5 | 26 |
| **42** | Mobio | Nasopharynx | 1.28E-01 | 4 | 4243 | 1367 | 1367 | 1000 | 37 | 0.999 | 2.751734 | 0.102763 | 4 | 31 |
| **43** | Mobio | Nares | 6.52E-01 | 1 | 12631 | 4852 | 4817 | 1000 | 41 | 0.981 | 2.223168 | 0.185125 | 6 | 38 |
| **44** | Mobio | Nares | 1.71E-01 | 2 | 519 | 148 | 142 | - | - | - | - | - | 5 | 20 |
| **45** | Mobio | Nares | 5.77E-01 | 3 | 13927 | 6336 | 6297 | 1000 | 60 | 0.968 | 2.065648 | 0.266236 | 9 | 58 |
| **46** | Mobio | Nares | 1.69E-01 | 4 | 4 | 1 | - | - | - | - | - | - | - | - |
| **47** | Mobio | Oropharynx | 5.33E+00 | 1 | 7822 | 3447 | 3436 | 1000 | 43 | 0.967 | 3.194208 | 0.078148 | 10 | 48 |
| **48** | Mobio | Oropharynx | 1.25E+01 | 2 | 6448 | 2880 | 2873 | 1000 | 74 | 0.982 | 2.382222 | 0.147249 | 7 | 34 |
| **49** | Mobio | Oropharynx | 8.20E+00 | 3 | 5105 | 2304 | 2286 | 1000 | 67 | 0.977 | 2.686964 | 0.153261 | 6 | 33 |
| **50** | Mobio | Oropharynx | 1.26E+01 | 4 | 6941 | 3153 | 3141 | 1000 | 48 | 0.975 | 2.397758 | 0.160779 | 7 | 34 |
| **51** | Mobio | Saliva dilution 1 | 2.63E+00 | 1 | 10869 | 4524 | 4524 | 1000 | 84 | 0.988 | 3.063447 | 0.11559 | 8 | 54 |
| **52** | Mobio | Saliva dilution 2 | 7.12E-01 | 1 | 11853 | 4964 | 4964 | 1000 | 50 | 0.994 | 3.007343 | 0.084945 | 6 | 39 |
| **Samples** | **DNA isolation method** | **Niche** | **qPCR (pg/ul)** | **Person** | **# reads/**  **sample** | **# maintained reads after filtering** | **# Unique sequences** | **# sequences after normalization** | **# Unique sequences** | **coverage** | **Npshannon**  **Diversity**  **index** | **Simpson**  **Diversity**  **index** | **#Taxa** | **# genera** |
| **53** | Mobio | Saliva dilution 3 | 1.26E-01 | 1 | 4983 | 2079 | 2079 | 1000 | 32 | 0.998 | 2.685894 | 0.109339 | 5 | 29 |
| **54** | Mobio | Saliva | 7.90E+02 | 1 | 4400 | 1756 | 1731 | 1000 | 94 | 0.958 | 2.859795 | 0.167407 | 7 | 38 |
| **55** | Mobio | Saliva | 1.44E+03 | 2 | 4142 | 1673 | 1636 | 1000 | 108 | 0.95 | 3.04428 | 0.113976 | 8 | 45 |
| **56** | Mobio | Saliva | 8.76E+02 | 3 | 4731 | 1877 | 1826 | 1000 | 117 | 0.925 | 3.170016 | 0.133213 | 7 | 44 |
| **57** | Mobio | Saliva | 7.17E+02 | 4 | 6788 | 2961 | 2940 | 1000 | 63 | 0.966 | 2.343327 | 0.215147 | 7 | 38 |
| **58** |  | PCR blank |  |  | 765 | 248 | 239 | - | - | - | - | - | 4 | 27 |
| **59** | Qiagen | Nasopharynx | 2.05E-01 | 1 | 11001 | 4157 | 4083 | 1000 | 109 | 0.953 | 3.096532 | 0.109477 | 10 | 92 |
| **60** | Qiagen | Nasopharynx | 9.60E-02 | 2 | 1434 | 333 | 304 | - | - | - | - | - | 9 | 48 |
| **61** | Qiagen | Nasopharynx | 5.12E-01 | 3 | 12310 | 5813 | 5775 | 1000 | 47 | 0.979 | 1.458908 | 0.402436 | 5 | 52 |
| **62** | Qiagen | Nasopharynx | 2.22E-01 | 4 | 6140 | 2193 | 2125 | 1000 | 105 | 0.946 | 3.123515 | 0.1624 | 10 | 82 |
| **63** | Qiagen | Nares | 3.00E-01 | 1 | 11494 | 4443 | 4368 | 1000 | 61 | 0.977 | 2.357297 | 0.160657 | 9 | 56 |
| **64** | Qiagen | Nares | 7.88E-02 | 2 | 2412 | 768 | 732 | - | - | - | - | - | 9 | 59 |
| **65** | Qiagen | Nares | 1.04E-01 | 3 | 12004 | 5468 | 5425 | 1000 | 53 | 0.973 | 1.815374 | 0.300294 | 8 | 63 |
| **66** | Qiagen | Nares | 7.60E-02 | 4 | 10949 | 4030 | 3961 | 1000 | 88 | 0.955 | 3.219733 | 0.093369 | 9 | 85 |
| **67** | Qiagen | Oropharynx | 5.47E+00 | 1 | 6929 | 3069 | 3045 | 1000 | 68 | 0.97 | 2.912286 | 0.106819 | 9 | 44 |
| **68** | Qiagen | Oropharynx | 2.52E+00 | 2 | 6063 | 2685 | 2672 | 1000 | 52 | 0.977 | 2.140906 | 0.231477 | 7 | 32 |
| **69** | Qiagen | Oropharynx | 4.38E+01 | 3 | 5097 | 2189 | 2167 | 1000 | 68 | 0.966 | 2.476902 | 0.187664 | 7 | 36 |
| **70** | Qiagen | Oropharynx | 8.54E+00 | 4 | 6236 | 2710 | 2692 | 1000 | 48 | 0.979 | 2.42874 | 0.134316 | 7 | 36 |
| **71** | Qiagen | Saliva dilution 1 | 4.80E-01 | 1 | 8028 | 3372 | 3372 | 1000 | 62 | 0.981 | 2.746838 | 0.113359 | 7 | 43 |
| **72** | Qiagen | Saliva dilution 2 | 1.10E+00 | 1 | 8610 | 3931 | 3931 | 1000 | 56 | 0.985 | 2.644317 | 0.115401 | 7 | 47 |
| **73** | Qiagen | Saliva dilution 3 | 1.64E-02 | 1 | 10851 | 4837 | 4837 | 1000 | 75 | 0.986 | 3.145416 | 0.087616 | 6 | 62 |
| **74** | Qiagen | Saliva | 1.03E+03 | 1 | 4731 | 1715 | 1681 | 1000 | 97 | 0.959 | 2.96414 | 0.114286 | 7 | 42 |
| **75** | Qiagen | Saliva | 1.47E+02 | 2 | 929 | 48 | 48 | - | - | - | - | - | 5 | 12 |
| **76** | Qiagen | Saliva | 8.53E+02 | 3 | 4889 | 1908 | 1863 | 1000 | 105 | 0.936 | 2.994282 | 0.121051 | 7 | 43 |
| **77** | Qiagen | Saliva | 1.22E+03 | 4 | 5762 | 2397 | 2367 | 1000 | 62 | 0.972 | 2.149457 | 0.226541 | 7 | 37 |
