# Supplementary material for: Deep Sequencing Analyses of Low Density Microbial Communities: Working at the Boundary of Accurate Microbiota Detection
Source: PLoS One. 2012 Mar 6;7(3):e32942. doi: 10.1371/journal.pone.0032942 (PMC3295791; doi:10.1371/journal.pone.0032942)
Supplement: Table S3 — Number of sequences that were able to be classified per taxonomic level. (DOC) [file pone.0032942.s009.doc]

**Table S3. Number of sequences classified per taxonomic level**

| **Taxonomic**  **level** | **Classification** | **# of sequences** |
| --- | --- | --- |
| **Phylum** | Assigned | 196982 |
|  | unassigned | 382 |
| **Class** | Assigned | 195810 |
|  | unassigned | 1554 |
| **Order** | Assigned | 195592 |
|  | unassigned | 1772 |
| **Family** | Assigned | 189268 |
|  | unassigned | 8096 |
| **Genus** | Assigned | 184003 |
|  | unassigned | 13361 |
