# Supplementary material for: Deep Sequencing Analyses of Low Density Microbial Communities: Working at the Boundary of Accurate Microbiota Detection
Source: PLoS One. 2012 Mar 6;7(3):e32942. doi: 10.1371/journal.pone.0032942 (PMC3295791; doi:10.1371/journal.pone.0032942)
Supplement: Methods S1 — More detailed information on sequence preprocessing and filter regimen. (DOC) [file pone.0032942.s010.doc]

**Methods S1**

**S1. Sequence preprocessing**
FASTA-formatted sequences and corresponding quality scores were extracted from the .sff data file generated by the GS-FLX-Titatium sequencer using the GS Amplicon software package (Roche, Branford, CT). Sequence data was processed using modules implemented in the Mothur v. 1.20.0. software platform . Due to the unique barcodes, sequences were binned by sample of origin. For further downstream analyses, barcodes and primer sequences were trimmed and low quality reads (containing ambiguous base calls, <1 error in the primer, ≤1 error in the barcode, <8 homopolymers anywhere in the sequence, the occurrence of a 50 nucluotide window with a qwindow average below 35, or a length >500 bp or <200 bp) were removed from the analyses. The data set was simplified by using the “unique.seqs” command to generate a non-redundant (unique) set of sequences. Unique sequences were aligned using the “align.seqs” command and an adaptation of the Bacterial SILVA SEED database as a template (available at: http://www.mothur.org/wiki/Alignment_database). In order to ensure that we were analyzing comparable regions of the 16S rRNA gene across all reads, sequences that started before the 2.5-percentile or ended after the 97.5-percentile in the alignment were filtered. Sequences were denoised using the “pre.cluster” command. This command applies a pseudo-single linkage algorithm with the goal of removing sequences that are likely due to pyrosequencing errors A total of 11814 potentially chimeric sequences were detected and removed using the “chimera.slayer” command . High quality aligned sequences were classified using the RDP-II naïve Bayesian Classifier. Aligned sequences were clustered into OTUs (defined by 97% similarity) using the average linkage clustering method. For each sample rarefaction curves were plotted.
