# Supplementary material for: Deep Sequencing Analyses of Low Density Microbial Communities: Working at the Boundary of Accurate Microbiota Detection
Source: PLoS One. 2012 Mar 6;7(3):e32942. doi: 10.1371/journal.pone.0032942 (PMC3295791; doi:10.1371/journal.pone.0032942)
Supplement: Methods S2 — MIQE form: Reporting requirement for Quantitative PCR Assays. (DOC) [file pone.0032942.s011.doc]

**Methods S2. MIQE: Reporting requirement for Quantitative PCR Assays**

**1.Administrative information**
A) Experiment description:
*Quantify 16S rDNA content in the environmental samples*

B) Responsible person and contact details
*G.Biesbroek, Research Group Microbiology and Systems Biology, TNO Earth, Environmental and Life Sciences, Zeist, The Netherlands:* [giske.biesbroek@tno.nl](mailto:giske.biesbroek@tno.nl)*, g.biesbroek@umcutrecht.nl*

**Sample annotation**
A) Sample description
Sample ID: *Material from human mucosal structures, e.g saliva, nasopharyngeal, oropharyngeal and nares swabs*Sample description: *by extraction method, niche, person and if in the dilution series by dilution* Template quantity used: *100 fentogram/µl to 1 nanogram/µl*

B) Sample role in qPCR assay
Sample type: *saliva spiked with oral bacteria: standard/reference samples
other samples are material from human mucosal structures e.g saliva, nasopharyngeal, oropharyngeal and nares swabs*
Inter run calibrator (*false*)
Calibrator sample (true)

**Target annotation**
A) Target description
Target ID: *16S rRNA gene*
 Sequence of primers: *16S Forward1 (5’-CGAAAGCGTGGGGAGCAAA-3’) and 16S reverse primer I
(5’-GTTCGTACTCCCCAGGCGG-3’) and 16S probe I (FAM-AAAAGATACCCTCGTAGT-MGB)*

B)Target role in qPCR assay
Target type*: target of interest*

**Thermal Cycling Conditions Information**
A) PCR program:
*PCR mix*: *12,5 µl Diagnode universal Mastermix (Hotstart Taq NA polymerase, optimized reaction buffer, 4mM MgCl2 and dNTPs), 1 µl of each primer (10µM), 1 µl of the probe (5µM), 6,5 µl of DNA free water and 3 µl of template DNA.*
*Thermal cycling conditions*: *initial DNA denaturation step 2 min 50 degrees Celsius and 10 min 95 degrees Celsius, followed by 40 cycles of 15 sec 95 degrees Celsius, 1 minute 60 degrees Celsius.*B) PCR format: *96-well plate A1-G12*

**Run data**
A) Instrument information
*7500 Fast Real-Time PCR system, Applied Biosystems, cataloguenr 4351107, Foster City, CA 94404 USA.
Sequence detection software version 1.4.0.25, 7500 Fast System SDS software*

B) Information required for each well: *on request*

**Software requirements**
RDML-Support
Software solutions, including databases, must support the import and export of RDML files: *true*qPCR machines must allow the export of raw data for the amplification as well as for melting curves: *true*
